# Genetic diversity and C2-like subgenogroup strains of enterovirus 71, Taiwan, 2008

**DOI:** 10.1186/1743-422X-7-277

**Published:** 2010-10-20

**Authors:** Yuan-Pin Huang, Tsuey-Li Lin, Li-Ching Hsu, Yu-Ju Chen, Yin-Hsin Tseng, Chiu-Chu Hsu, Wen-Bin Fan, Jyh-Yuan Yang, Feng-Yee Chang, Ho-Sheng Wu

**Affiliations:** 1 Research and Diagnostic Center, Centers for Disease Control, Department of Health, Taipei, Taiwan, R.O.C; 2 School of Medical Laboratory Science and Biotechnology, Taipei Medical University, Taipei, Taiwan, R.O.C

## Abstract

**Background:**

Human enterovirus 71 (EV-71) is known of having caused numerous outbreaks of hand-foot-mouth disease, and other clinical manifestations globally. In 2008, 989 EV-71 strains were isolated in Taiwan.

**Results:**

In this study, the genetic and antigenic properties of these strains were analyzed and the genetic diversity of EV-71 subgenogroups surfacing in Taiwan was depicted, which includes 3 previously reported subgenogroups of C5, B5, and C4, and one C2-like subgenogroup. Based on the phylogenetic analyses using their complete genome nucleotide sequences and neutralization tests, the C2-like subgenogroup forms a genetically distinct cluster from other subgenogroups, and the antisera show a maximum of 128-fold decrease of neutralization titer against this subgenogroup. In addition, the subgenogroup C4 isolates of 2008 were found quite similar genetically to the Chinese strains that caused outbreaks in recent years and thus they should be carefully watched.

**Conclusions:**

Other than to be the first report describing the existence of C2-like subgenogroup of EV-71 in Taiwan, this article also foresees a potential of subgenogroup C4 outbreaks in Taiwan in the near future.

## Background

Belonging to the genus *Enterovirus *of the family *Picornaviridae*, human enterovirus 71 (EV-71) is one of the most causative pathogens infecting humans and may cause outbreaks of hand-foot-mouth disease (HFMD), herpangina, and severe neurological symptoms, especially in young children [1]. There are over one hundred serotypes identified in the genus *Enterovirus *[2], which was originally classified into polioviruses, coxsackievirus A, coxsackievirus B, and echoviruses on the basis of differences in cell tropism, infectivity, antigenicity, and pathogenicity [1]. In recent years, the genus *Enterovirus *was re-classified into ten species, *Human enterovirus A*, *Human enterovirus B*, *Human enterovirus C*, *Human enterovirus D*, *Simian enterovirus A*, *Bovine enterovirus*, *Porcine enterovirus B*, *Human rhinovirus A*, *Human rhinovirus B*, and *Human rhinovirus C *based on the molecular characteristics. Former Coxsackievirus A2 (CV-A2), CV-A3, CV-A4, CV-A5, CV-A6, CV-A7, CV-A8, CV-A10, CV-A12, CV-A14, CV-A16, EV-71, EV-76, EV-89, EV-90, EV-91, EV-92, Simian enteroviruses SV19, SV43, SV46, and A13 are now members of *Human enterovirus A *[3-5].

The positive-stranded RNA genome of EV-71 possesses approximately 7,500 nucleotides and includes three genomic regions designated P1, P2, and P3. P1 region encodes four structural capsid proteins (VP4, VP2, VP3, and VP1), while P2 and P3 encodes seven nonstructural proteins (2A, 2B, 2C, 3A, 3B, 3C, and 3D). The nonstructural proteins are involved in polyprotein processing, and the capsid proteins, especially VP1, contain many neutralization antigenic sites and correspond to the virus serotyping [6]. In previous studies, the N-terminal portion of the VP1 capsid protein (composed of 297 amino acids) was likely to contain a major antigenic region and had important neutralizing antibody determinants [7,8]. But in another study, two synthetic peptides containing the C-terminal part of the VP1 protein (amino acid 163-177 and 208-222) were capable of eliciting neutralizing antibodies against EV-71 [9]. In addition, three regions on the VP1 protein (amino acid 66-77, 145-159, and 247-261) were identified to be capable of inducing human EV-71-specific CD4^+ ^T-cell proliferation [10]. However, the accurate locations of neutralizing epitopes are still uncertain. Recombination found in the same serotype (intratypic) or in the different serotype (intertypic) and point mutation events result in the evolution of EV. Multiple strains circulating at the same area may increase the possibility of recombination, and many recombinants have been observed in EV [11-13].

EV-71 is genetically divided into three genogroups, A, B, and C, on the basis of the VP1 sequences analyses [14]. Genogroups B and C are each further divided into five subgenogroups, designated as B1-B5 and C1-C5, while genogroup A contains only one strain, the prototype strain BrCr [15,16]. In addition, some uncommon subgenogroups were also identified. For instance, isolates of subgenogroups B0 were first observed in The Netherlands in 1963 [17], and those of subgenogroup C0 were observed in Japan in 1978 [18,19]. One Indian isolate in 2001 was genetically distinct from all other EV-71 strains and designated as genotype D [20].

Since EV-71 was first isolated in California in 1969, many EV-71 outbreaks have been reported worldwide, for instance, several outbreaks took place in the USA, Japan, and other countries in the 1970s (subgenogroup B1), in Hong Kong, Australia, and the USA in the 1980s (subgenogroups B1, B2, and C1), and especially in the Asian Pacific region in recent years [21,22]. Subgenogroup B3 was described in Sarawak, Singapore, and Australia in 1997, 1998, and 1999, respectively, while subgenogroup C4 was identified on Mainland China in 1998. After that, EV-71 epidemics of subgenogroup B4 were reported in Singapore, Sarawak, and Sydney, and those of subgenogroup C3 were described in Korea in 2000 [15]. Subgenogroup B5 was identified in Sarawak, Japan, and Singapore in the last decade and subgenogroup C5 in southern Vietnam in 2005 [16]. Since one subgenogroup could be found from different countries in the same or different period, to predict the epidemiological pattern of EV-71 infections is not easy. For example, subgenogroup C1 was first described in the United States in 1986 [14], but caused several outbreaks in Germany, Australia, the United Kingdom and other countries [23-25]. On the other hand, one subgenogroup could be identified in the same area during a long period; for instance, subgenogroup C4 showed up repeated on Mainland China from 1998 to 2008 [26].

In Taiwan, a large outbreak was reported in 1998, followed by two lesser outbreaks in 2000 and 2001, and one more in 2008 [27-29]. Based on a study covering 8-years, the incidence of mild cases of HFMD/herpangina was reported as 0.8 to 19.9 cases per sentinel physician per week. The seasonal incidence varied, but usually peaked in the summer [30]. Over the past several years, co-circulation patterns of various genetic subgenogroups were frequently observed in Taiwan. Back in 1998 for instance, the subgenogroup C2 was found to be the major one with subgenogroups B4 and C4 as two minors. Afterwards, the subgenogroup B4 was singled out as the major cause of the outbreaks with C4 as a minor in 2002, and then subgenogroup B5 became the major one with a minor C5 from 2006 to 2008 [21,31]. In such a situation, it is expected that the possibility of recombination between various subgenogroups of EV-71 increases. Therefore, we persistently analyzed all EV-71 isolates collected by our surveillance system, and tried to find out if any isolates were genetically distinct from those EV-71 strains isolated from earlier outbreaks by phylogenetic analyses and neutralization tests.

## Results

### Epidemiological results

According to our laboratory surveillance data, EV-71 viruses of various subgenogroups were isolated from 989 patients in Taiwan in 2008. They were 413 females, 564 males, and 12 with gender not specified, and no significant differences were observed in gender distribution (p > 0.05). Among these patients with age ranging from 1 week to 38 years old, most (810/989, 81.9%) were under 5, including 342 girls, 460 boys and 8 with missing data of gender. EV-71 infections were reported throughout the year with a peak in the summer, roughly between May and July.

### Basic Local Alignment Search Tool (BLAST) result

**Figure 1:**
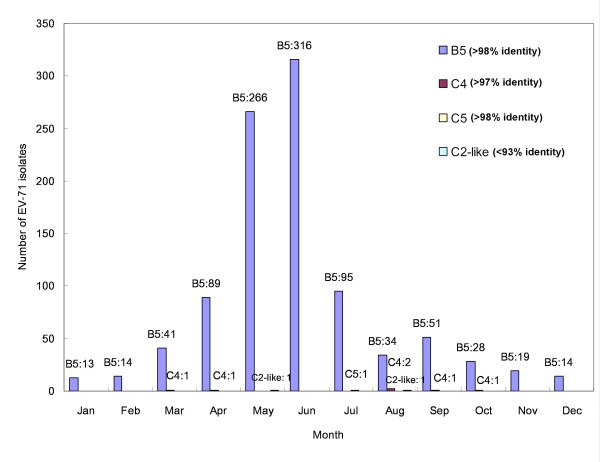
**Different subgenogroups of 989 enterovirus 71 (EV-71) isolates in Taiwan in 2008 according to the BLAST results**. The subgenogroup was determined by BLAST analysis of partial VP1 region nucleotide sequences. There were 980 subgenogroup B5 isolates, 6 subgenogroup C4 isolates, 1 subgenogroup C5 isolate, and 2 subgenogroup C2-like isolates identified according to the BLAST analysis.

Four subgenotypes of EV-71, including 980 subgenogroup B5 isolates, 6 subgenogroup C4 isolates, 1 subgenogroup C5 isolate, and 2 subgenogroup C2-like isolates, were identified according to the BLAST results of partial VP1 region nucleotide sequences (Figure 1). All isolates showed extremely high identities with their respective reference strains (>97%), except the two C2-like isolates (<93%). The genotyping of the subgenogroup C2-like isolates were thus further confirmed by phylogenetic analysis. These two isolates, 2008-07776 and 2008-00643, were collected in Taipei County in May and August, respectively.

### Phylogenetic analysis and recombination analysis

**Figure 2:**
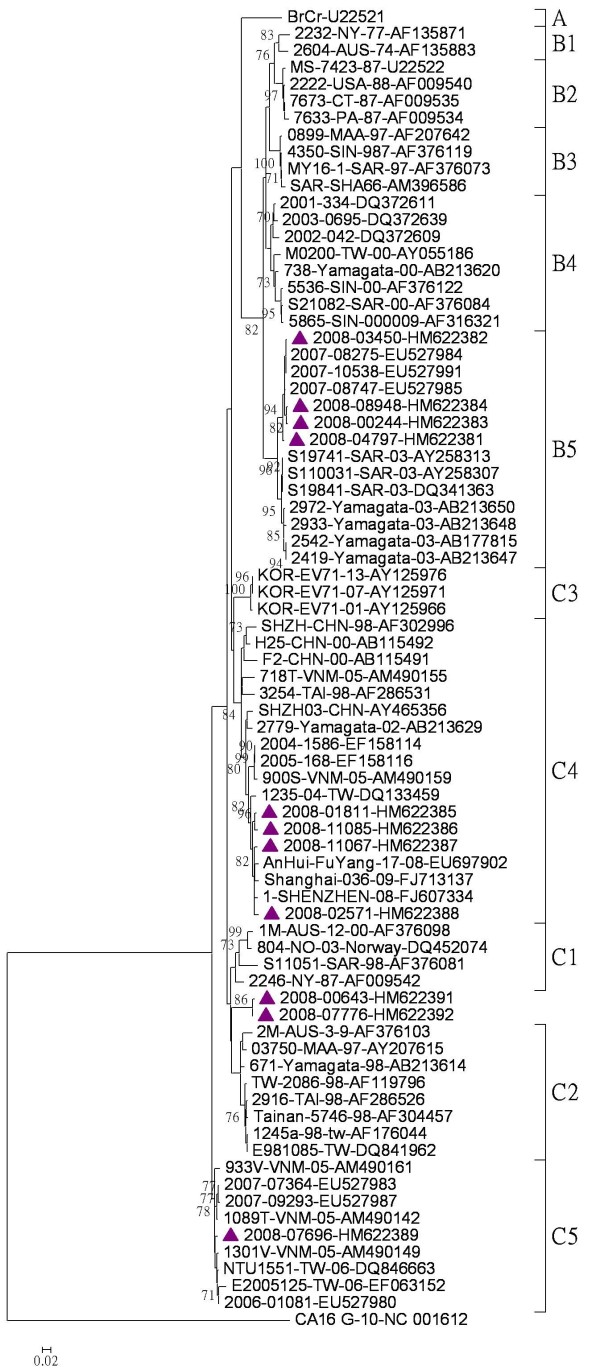
**Phylogenetic analysis of enterovirus 71 strains based on partial VP1 gene sequence (nucleotide position 16-418)**. Phylogenetic analysis was performed based on partial VP1 gene nucleotide sequences of reference strains from the GenBank and 11 representative isolates chosen from 989 sequenced isolates from Taiwan in 2008. The phylogenetic tree was constructed by the neighbor-joining method with *MEGA *version 4 software, and the reliabilities indicated at the branch nodes were evaluated using 1,000 bootstrap replications. Only values of over 70% were shown. The prototype coxsackievirus A16 (CA16) G-10 strain was used as an out-group.

After the BLAST process, four subgenogroup B5 and four subgenogroup C4 isolates randomly chosen, along with the only one subgenogroup C5, and two subgenogroup C2-like isolates, were used in a phylogenetic analysis on partial VP1 gene nucleotide sequence (Figure 2). The B5 and C5 isolates turned out to be genetically similar to the Taiwan strains isolated in 2007, while the C4 isolates tested were close to those China strains isolated in 2008-2009. Besides, the C2-like isolates were located in genogroup C, but not within any known subgenogroup.

**Figure 3:**
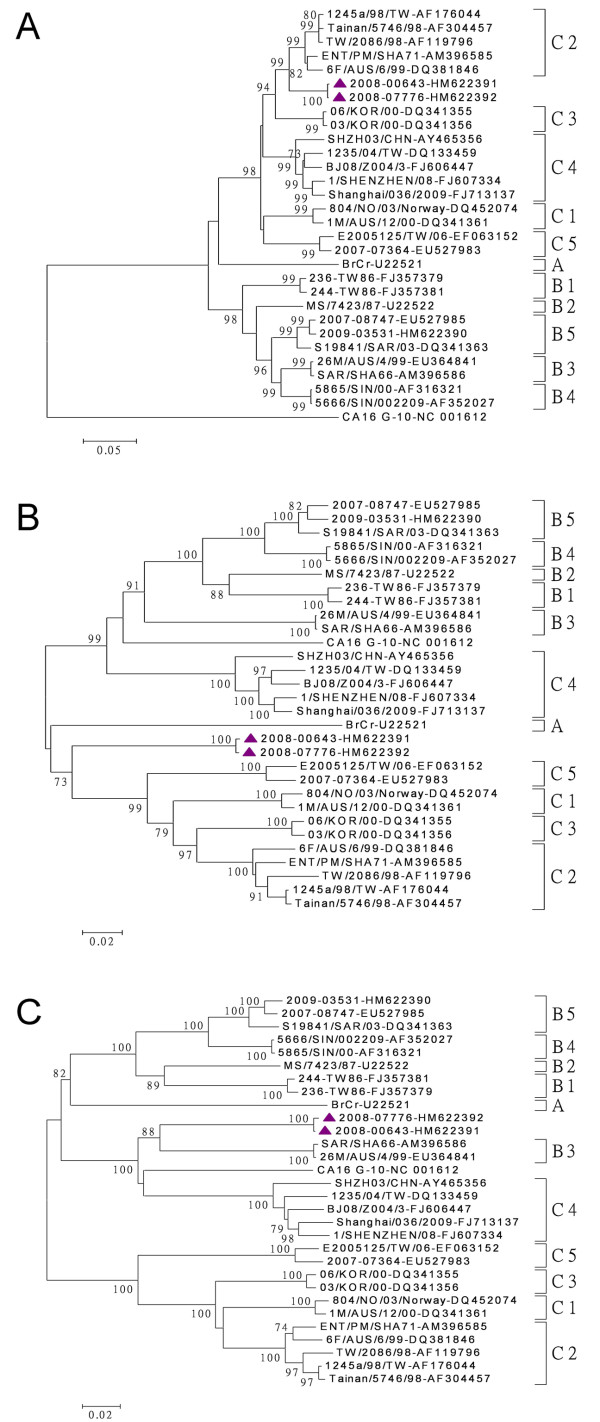
**Phylogenetic analyses of enterovirus 71 strains**. The phylogenetic tree was constructed by the neighbor-joining method with *MEGA *version 4 software, and the reliabilities indicated at the branch nodes were evaluated using 1,000 bootstrap replications. Only values of over 70% were shown. The prototype coxsackievirus A16 (CA16) G-10 strain was used as an out-group. The tree was drawn on the basis of the P1 region nucleotide sequences (A), the P2 region nucleotide sequences (B), and the P3 region nucleotide sequences (C).

Due to the uncertain genotyping on partial VP1 gene, with no more than 93% of the nucleotide identity between the C2-like isolates and the reference strains, each gene region of these two isolates was further sequenced and recombination analyses conducted. The nucleotide and amino acid identities between EV-71 subgenogroups were presented in Table 1. No amino acid changes were observed for C2-like isolates in the two regions of VP1 protein which were capable of eliciting neutralizing antibodies (amino acids 163-177 and 208-222). Moreover, there were no unique changes in three regions of VP1 protein, which were capable of inducing human EV-71-specific CD4^+ ^T-cell proliferation (amino acids 66-77, 145-159, and 247-261). The phylogenetic analysis results showed that these 2 subgenogroup C2-like isolates formed a distinct cluster within genogroup C based on P1 and P2 region nucleotide sequences (Figure 3, panels A-B), and within genogroup B based on P3 region nucleotide sequences (Figure 3, panel C). The phylogenetic trees of each gene sequences were shown in Additional File 1.

**Table 1:** Percent identity (%) of nucleotide and amino acid sequences in different gene fragment between subgenogroup C2-like and other subgenogroups of enterovirus 71*

Sub- genogroup		Gene												
		
		5'-UTR	VP4	VP2	VP3	VP1	2A	2B	2C	3A	3B	3C	3D	Complete
A	nt	83.1	84.0	80.8-81.1	83.0-83.3	83.8	79.1	76.0	78.3	77.9-78.2	72.7	76.5	76.8	79.3-79.4
	aa		100	97.6	97.9	94.9	96.6	92.9	96.6	97.6	90.9	93.4	91.7	95.3
B1	nt	84.2-84.4	78.7	84.5-84.6	79.6-79.8	83.5-83.7	76.8-78.0	72.3-73.0	82.4-82.5	81.3-81.7	81.8	79.5-80.5	77.7-78.3	80.2-80.6
	aa		98.5-100	97.6	97.5	96.9-97.3	94.0-94.6	91.9-92.9	95.1-95.4	96.5	95.4	96.7	93.2-94.3	95.8-95.9
B2	nt	85.1-85.2	80.1	84.1	80.3-80.5	83.3-83.6	79.1	73.4	82.6	78.6-79.0	80.3	78.5	78.3-78.4	80.3-80.5
	aa		100	97.6	97.1	97.3	94.6	92.9	94.5	97.6	95.4	96.7	93.9-94.1	95.8
B3	nt	84.1-84.6	82.6-83.0	82.4-82.5	79.2-79.6	82.9-83.3	78.8-79.1	76.7	83.4-83.6	83.7-84.4	90.9	85.4-85.6	84.7-85.2	82.4-82.6
	aa		100	97.6-98.0	96.6	96.9	95.3	94.9	97.5-97.8	98.8	95.4	98.3	96.5-97.4	97.0-97.3
B4	nt	83.9-84.5	83.0	83.3-83.4	80.3-80.4	83.3-83.6	80.6	75.0	82.3-82.6	79.0-79.8	83.3	78.6-78.8	78.2-78.5	80.4-80.6
	aa		100	98.0	97.1	97.6	95.3	92.9	96.3	95.3-96.5	100	95.6	94.5-94.8	96.2-96.3
B5	nt	79.8-83.4	82.6-83.5	81.7-82.8	80.9-82.3	83.6-84.5	80.6-81.7	73.0-73.7	82.1-82.8	79.4-81.0	84.8-86.3	77.7-78.1	77.5-78.1	80.3-80.5
	aa		100	97.2-98.0	97.1	97.9-98.3	94.0-95.4	92.9	95.7-96.3	97.6	95.4-100	95.0-96.1	94.1-94.5	96.1-96.3
C1	nt	81.6-82.2	88.4	88.5-88.9	88.2-89.6	88.4-89.0	85.5-86.2	84.8-85.1	79.7-79.9	75.9-77.1	77.2-78.7	75.2-75.5	79.4-79.9	82.4-83.0
	aa		98.5-100	99.2-99.6	99.1-99.5	99.6	96.6-97.3	94.9	96.6-96.9	91.8	95.4	93.4-93.9	94.3-94.5	96.7-96.9
C2	nt	81.2-83.3	91.7-94.6	93.7-94.4	93.8-95.1	91.9-93.0	90.8-92.6	90.9-91.5	78.7-80.4	77.1-79.0	75.7-77.2	73.2-75.0	78.9-80.0	84.0-85.4
	aa		100	99.2-100	100	98.6-99.6	98.6	91.9-93.9	91.1-96.9	87.2-93.0	95.4	87.4-93.9	93.2-94.3	95.1-96.9
C3	nt	83.2-83.5	87.9	90.4-90.5	90.4-91.0	88.1-88.6	86.8-87.1	88.5	78.8-79.0	77.1-77.9	75.7	75.4-75.7	78.5-78.9	82.9-83.2
	aa		100	99.6	100	99.6	96.6-97.3	93.9	97.2	93.0	95.4	92.3-92.8	93.9-94.1	96.8
C4	nt	83.1-84.1	88.4-90.8	88.7-90.6	89.2-90.4	86.1-87.9	82.6-84.0	73.7-75.4	82.4-83.5	80.2-81.3	83.3-86.3	83.0-83.7	81.8-83.6	83.8-84.4
	aa		97.1-100	99.6-100	98.7-99.5	98.6-99.6	95.3-96.0	92.9-94.9	97.2-98.4	97.6-98.8	95.4	96.1-97.2	95.4-96.5	97.4-97.9
C5	nt	82.5-82.9	89.3-90.3	88.0-88.7	87.1-87.4	87.8	86.2-86.8	82.4-83.1	78.3-79.3	74.0-74.8	80.3-81.8	76.3-76.5	77.3-77.4	81.4-82.5
	aa		100	99.6	100	98.6-99.6	96.0	91.9-93.9	97.2	93.0-94.1	95.4	93.4-93.9	93.2-93.9	96.6-96.7

*Subgenogroup A: BrCr-CA-70 (GenBank accession no. U22521), B1: 236-TW86 (FJ357379) and 244-TW86 (FJ357381), B2: 26M/AUS/4/99 (EU364841), B3: SAR/SHA66 (AM396586) and 26M/AUS/4/99 (EU364841), B4: 5865/SIN/000009 (AF316321) and 5666/SIN/002209 (AF352027), B5: S19841-SAR-03 (DQ341363), 2007-08747 (EU527985) and 2009-03531 (HM622390), C1: 804/NO/03 (DQ452074) and 1M-AUS-12-00 (DQ341361), C2: 1245a/98/TW (AF176044), ENT/PM/SHA71 (AM396585), Tainan/5746/98 (AF304457), TW/2086/98 (AF119796) and 6F/AUS/6/99 (DQ381846), C3: 06/KOR/00 (DQ341355) and 03/KOR/00 (DQ341355), C4: 1235/04/TW (DQ133459), BJ08/Z004/3 (FJ606447), 1/SHENZHEN/08 (FJ607334), Shanghai/036/2009 (FJ713137) and SHZH03-CHN (AY465356), C5: E2005125-TW (EF063152) and 2007-07364 (EU527983), C2-like: 2008-00643 (HM622391) and 2008-07776 (HM622392).

**Figure 4:**
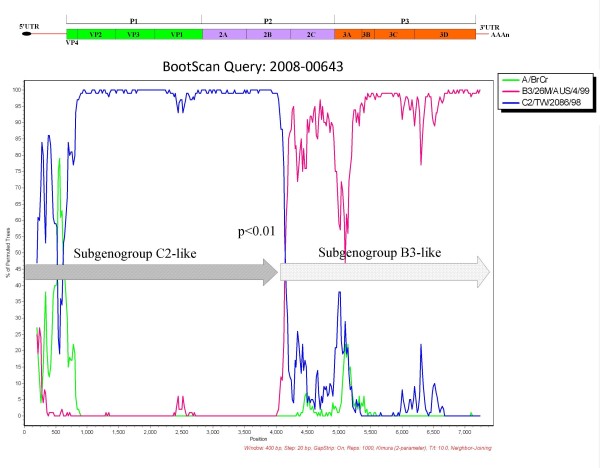
**Bootscan analyses of enterovirus 71 nucleotide sequences**. The subgenogroup C2-like strain 2008-00643 was queried against other subgenogroups of enterovirus 71 using SimPlot, version 3.5.1, in a sliding window of 400 nucleotides with a 20 nucleotides step.

One suspected recombination event was shown in the similarity plot and bootscan analyses between subgenogroup C2 and subgenogroup B3 of EV-71 (p < 0.01) (Figure 4).

### Preparation of anti-enterovirus rabbit serum, and neutralization test

Anti-EV-71 rabbit sera against three subgenogroups (C2, C5, and B5) of EV-71 virus, with 100 cell culture infective dose (CCID_50_) viruses per 50 μl for immunization, were used for neutralization test. Table 2 shows the neutralization antibody titers against different subgenogroups of EV-71. Based on the data against their homo-subgenogroup viruses, antisera C2, C5, and B5 showed a 2- to 16-fold decrease in titers against their hetero-subgenogroups. However, the result of neutralization antibody titers of the same antisera against the C2-like subgenogroup showed an obvious difference (p < 0.05), with an 8- to 128-fold decrease compared to those of their homo-subgenogroup.

**Table 2:** Neutralization antibody titers of rabbits antisera against enterovirus 71 (EV-71) from different subgenogroups

Antisera no.	Subgenogroup of immunogen	EV-71 strain					
		
		97111207 (C2) *	E2004104 (C4) *	E2006125 (C5) *	E2002042 (B4) *	E2007599 (B5) *	C2-like
1		8,192	1,024	1,024	4,096	4,096	256
2	C2	32,768	8,192	4,096	131,072	131,072	2,048
3		32,768	32,768	16,384	65,536	65,536	4,096

4		32,768	262,144	131,072	65,536	65,536	4,096
5		32,768	524,288	131,072	262,144	262,144	4,096
6	C5	65,536	524,288	262,144	262,144	262,144	8,192

7		8,192	262,144	32,768	32,768	32,768	2,048
8		16,384	8,192	32,768	262,144	131,072	2,048
9	B5	4,096	4,096	4,096	32,768	8,192	128
10		32,768	131,072	131,072	262,144	131,072	1,024

* Statistically significant difference in log_10_-transformed data when compared to subgenogroup C2-like group (p < 0.05).

In addition, there were 11 pairs of serum samples used for neutralization test in this study, including acute-phase serum (3-7 days post infection) and recovery-phase serum (15-39 days post infection) (Table 3). Sera obtained from the patients with EV-71 infection belonging to subgenogroups B4, C4, C5, and B5 showed a maximum of 16-fold decrease in neutralization titers against hetero-subgenogroups of EV-71 as compared to the ones against their homo-subgenogroup. On the contrary, sera showed a maximum of 128-fold decrease against the C2-like subgenogroup. Taken together, these results indicated a divergence of antigenic relationship between the subgenogroup C2-like and other subgenogroups.

**Table 3:** Serum neutralization antibody titers against different subgenogroups of enterovirus 71 (EV-71)

Antisera no.	Subgenogroup of EV-71 infection	Sampling period (days post infection)*		EV-71 strain					
				
				97111207 (C2) *	E2004104 (C4) *	E2006125 (C5) *	E2002042 (B4) *	E2007599 (B5) *	C2-like
1	B4	AP	(6)	1,024	1,024	1,024	1,024	2,048	16
		RP	(32)	1,024	1,024	1,024	1,024	2,048	64
2	C4	AP	(5)	64	1,024	256	1,024	512	32
		RP	(13)	512	1,024	1,024	1,024	4,096	64
3	C5	AP	(5)	1,024	2,048	2,048	1,024	1,024	64
		RP	(16)	1,024	1,024	4,096	2,048	512	256
4	C5	AP	(4)	256	512	1,024	2,048	2,048	16
		RP	(16)	256	1,024	1,024	1,024	1,024	32
5	C4	AP	(4)	128	1,024	512	2,048	2,048	8
		RP	(17)	512	4,096	2,048	16,384	16,384	128
6	B5	AP	(6)	256	512	512	512	1,024	32
		RP	(27)	2,048	4,096	2,048	4,096	4,096	256
7	B5	AP	(7)	64	32	32	64	64	<8
		RP	(22)	128	128	256	512	1,024	64
8	B5	AP	(3)	8	32	16	64	128	<8
		RP	(15)	1,024	8,192	4,096	4,096	4,096	64
9	B5	AP	(4)	32	64	64	64	32	16
		RP	(39)	128	128	256	512	256	64
10	B5	AP	(4)	1,024	512	512	512	1,024	64
		RP	(23)	512	256	256	1,024	512	64
11	B5	AP	(4)	128	512	256	256	1,024	32
		RP	(16)	2,048	8,192	4,096	4,096	8,192	256

* Statistically significant difference in log_10_-transformed data when compared to subgenogroup C2-like group (p < 0.05). AP: acute phase; RP: recovery phase.

## Discussion

Enterovirus infections, especially EV-71, were associated with HFMD, herpangina, and neurological diseases and very common in the West Pacific region where Taiwan locates. There has been about two thousands isolates in Taiwan reported by the surveillance program each year since 2001 [16,32]. Moreover, emergence of new EV-71 subgenogroups was reported continuously. Because the VP1 gene is highly related to host neutralization antibodies and viral virulence, determining the genogroup of EV-71 is generally based on the VP1 gene sequence [17], and three genotypes were recognized accordingly [14]. A combination of VP1 and 3D gene sequences was proposed to be used for initial genotyping [19]. However, only a few studies about the antigenic variances of EV-71 have been reported [29,33].

In this study, we reported a genetic and antigenic diversity of EV-71 subgenogroups in Taiwan in 2008, including 3 previously reported subgenogroup C5, B5, C4, and one C2-like subgenogroup. The surveillance results of EV-71 molecular epidemiology in Taiwan was quite different from those in other counties, for example, genogroup C was the only one spotted in the United Kingdom from 1998 to 2006 and in Germany from 1997 to 2007 [24,25].

EV-71 of subgenogroup C5 was first isolated in southern Vietnam in 2005 and caused an outbreak with neurological disease and high prevalence [16]. According to our surveillance data in 2008, the isolate of subgenogroup C5 was identified in July, and this subgenogroup was still recognized in 2009 (unpublished data). Although these subgenogroup C5 strains were in low numbers and did not result in outbreaks in Taiwan in recent years [21], a previous report of EV-71 showed that the genogroups which caused outbreaks were usually in circulation 2 to 5 years before the onset of the outbreaks [29]. Hence we could not exclude the possibility of an outbreak caused by subgenogroup C5 strains in the subsequent years. Subgenogroup B5 strains were isolated in Taiwan in 2003 and 2007, and became the dominant genogroup in outbreaks in 2008. The antigenic variation of subgenogroup B5 strains had been discussed previously [21,29], and B1/B4, B5, and C2/C4 were divided into different groups in the antigenic map. But in another study, subgenogroup B5 was proposed to be redesignated as B4 based on the genetic analysis of complete genome nucleotide sequences [19]. More studies are needed to explain the inconsistent results between antigenic and genetic typing.

Subgenogroup C4 circulated and evolved in neighbouring countries in recent years chronologically, especially in China. There were two clusters of subgenogroup C4 strains in China from 1998 to 2008, C4b (from 1998 to 2004) and C4a (from 2003 to 2008), and the Shandong C4a strains were further divided into three lineages [26]. In Taiwan, subgenogroup C4 was first isolated in 1998 (as C4b cluster in China), and then caused outbreaks from 2004 to 2005 (as C4a cluster in China) [31]. According to the sequence analyses in this study, we identified several C4 isolates which were correlated well with C4 strains in China in 2008-2009, but not correlated with those isolated in Taiwan in 2004-2005, indicating that the virus was supposed to be transmitted from China (Figure 2). This subgenogroup caused several outbreaks in China over the last four years [26,34], but not in Taiwan, which was possibly due to herd immunity related to the subgenogroup C4 epidemic in Taiwan from 2004 to 2005. However, we still detected several subgenogroup C4 isolates in 2009 (unpublished data), and an increase of severe cases in early 2010, indicating that a potential of subgenogroup C4 outbreak in 2010 was expected, and to maintain a comprehensive surveillance system for enteroviruses seems to be a must.

Inter-genogroup, inter-subgenogroup and intra-subgenogroup average divergences of EV-71 complete genome nucleotide sequences were 17-22%, 10-14% and 1-10%, respectively [19]. However, further evidence is needed to designate the subgenogroup C2-like as a new subgenogroup. On the other hand, the lower neutralization antibody titers of subgenogroup C2-like (with a maximum of 128-fold decrease) indicated the antigenic differences with other subgenogroups (Table 2, Table 3). In previous study, a close antigenic relationship among the EV-71 isolates belonging to genogroups B and C was reported. The neutralization titers of the antisera for different genogroups of EV-71 ranged from 512 to >1,024, while the titers of the antisera for homologous EV-71 isolates were >1,024 [33]. The antigenic diversity of subgenogroup C2-like viruses displayed in this study may result in the inefficiency of herd immunity, and cause concerns on vaccine development for EV-71, e.g., monovalent or polyvalent vaccine. In addition, to further clarify the divergences, more researches using EV-71 monoclonal antibodies are needed for identification of neutralization epitopes.

The subgenogroup C2-like was supposed to be a recombinant originated from subgenogroup C2 and B3 based on a bootscan analysis. In addition, the subgenogroup C2-like viruses were isolated from different patients in different month, demonstrating that this subgenogroup was not a single case but circulated for a period of time. In Taiwan, subgenogroup C2 strains were only observed in 1998 [35], but subgenogroup B3 strains were never reported before. It is difficult to trace the actual spread route due to the recently more frequent international travel and fluxes of laborers. However, each gene region of the subgenogroup C2-like was 73.2-95.1% identical to that of other subgenogroups (Table 1), so it is supposed probably that the ancestors of this subgenogroup were imported into Taiwan before 2008, experienced recombination events, and then evolved into a unique subgenogroup. For enteroviruses, recombination was most reported to occur in the nonstructural protein region [36], while few reports demonstrated recombination in the structural capsid protein region [37]. The putative recombination breakpoint at 2B gene in this study was not reported yet. Other breakpoints at the 3'-termini of the 2A and 3C regions [38], 3D and 3'UTR regions [39] were identified in previous reports. It was speculated that the higher degree of similarity in nonstructural protein region may favor the occurrence of recombination. However, variants with recombination or deletion mutations, especially in structural protein region, may not survive or replicate less efficiently [13,40]. The subgenogroup C2-like strains showed lower CCID_50 _than other subgenogroups (data not shown), and it may explain why this subgenogroup did not cause outbreaks in 2008. Another possibility was that the prevalence of subgenogroup C2-like might be underestimated due to asymptomatic infections or mild illness despite a surveillance system had been set up.

## Conclusions

In summary, firstly, we described a genetic and antigenic diversity of EV-71 subgenogroups in Taiwan in 2008, including 3 previously reported subgenogroups C5, B5, and C4, and one C2-like subgenogroup. Secondly, the subgenogroup C4 isolates in 2008 were genetically similar to the Chinese strains causing outbreaks in recent years, so we need to closely monitor if these subgenogroup C4 outbreaks happen or not in Taiwan in the next few years. Thirdly, due to the diversity of phylogeny, rapid changing of subgenogroups, and the potential of severe and fatal outbreaks on their way, it is a must to monitor the recombination events as well as antigenic and genetic evolution of EV-71 very attentively and carefully.

## Methods

### Virus isolation and identification

EV-71 viruses used in this study were collected by the surveillance systems under Centers for Disease Control, Taiwan (Taiwan CDC). These 989 strains were isolated from throat swabs, stools, sera, or cerebrospinal fluid specimens taken from patients with HFMD, herpangina, and other symptoms related to enterovirus infection. Virus isolation was carried out using rhabdomyosarcoma (RD), human diploid fibroblast (MRC-5), African green monkey kidney (Vero), human lung carcinoma (A549), monkey kidney (LLC-MK2), or human epidermoid carcinoma (HEp-2) cell lines until cytopathic effects (CPE) were observed. The isolates were then identified by immunofluorescence assay (IFA) using an EV-71 commercial monoclonal antibody (Light Diagnostic, USA). The CCID_50 _of the virus was calculated by the Reed and Muench method [41].

### RT-PCR and Sequencing

Viral RNA was extracted according to the manufactory protocol from 140 μl of culture supernatant by QIAamp Viral RNA Mini Kit (Qiagen, Santa Clara, CA). One-step RT-PCR of VP1 gene was performed for all 989 EV-71 isolates with primer set 159/162 [14], and full-length RT-PCR was performed for two isolates (2008-07776 and 2008-00643) as described previously [13]. The products were confirmed by agarose electrophoresis and were stained with ethidium bromide. DNA was sequenced in both directions using BigDye Terminator Ready Reaction Cycle Sequencing Kit and an automated sequencer ABI 3730 (Applied Biosystems, Foster City, CA, USA).

### Sequence analysis and recombination analysis

Identification and subtyping was carried out by sequence comparisons with reference EV sequences in GenBank using the BLAST [42] and confirmed by phylogenetic analysis. The DNA sequences were assembled and then aligned with reference sequences using the Clustal W program by BioEdit (version 7.0.9.0) software [43]. Phylogenetic trees were constructed using the neighbor-joining method by *MEGA *version 4 software [44] with 1,000 replications of bootstrap analyses. The prototype coxsackievirus A16 (CA16) G-10 strain was used as an out-group. Detection of recombination events among the subgenogroups of EV-71 viruses using the full genome sequence was determined by similarity plot and bootscan analyses using SimPlot, version 3.5.1 [45] as previously described [21,46]. The nucleotide identity was calculated using the Kimura 2-parameter method with a transition-transversion ratio of 10 [47] and a sliding window of 400 nucleotides in 20 nucleotide steps. The recombination breakpoints were determined by the maximization of χ^2 ^analysis [48], and the p values for the resulting informative sites were calculated using the χ^2 ^test.

### Preparation of different subgenogroups EV-71 virus as immunogen for rabbits immunization

Three ancient EV-71 strains of subgenogroups C2, C5 and B5 (AFP98111207, E2006125, and E2007599, respectively) in Taiwan were selected for antiserum preparation. These strains were propagated in RD cells, and the CCID_50 _was determined before animal inoculations. Anti-enterovirus rabbit serum was prepared as described preciously [49]. Briefly, New Zealand White rabbits were immunized intravenously with 5 ml of UV-inactivated virus stock (>10^8 ^CCID_50_/ml) of above three subgenogroups of EV-71. The animals were subsequently boosted four times with the same dose at a 2-day interval, except with a double dose (10 ml) at the final boosting on day 42, and the sera were tested for neutralization antibodies on day 49.

### Determination of neutralization antibody titers

Rabbit antisera and pairs of serum samples collected during the acute-phase and recovery-phase from patients with EV-71 infection were examined for neutralization antibodies. All sample determinations were performed in duplicate. Sera were first inactivated at 56ºC for 30 min, and then diluted two-fold serially in DMEM from 1:8 to 1:1,024. One-hundred CCID_50 _viruses (50 μl) were added to the well contained above serially diluted antiserum, and the mixtures were then incubated in a CO_2 _incubator at 36ºC for 60 min. Later, 100 μl of RD cell suspension containing approximately 3 × 10^4 ^cells was added to each well, and the CPE was recorded during the next 4 days. The neutralization end-point titer is defined as the highest dilution fold at which 50% of cells showing complete inhibition of CPE formation.

### Statistical analysis

Differences between proportions were tested using the χ^2 ^test. The neutralization antibody titers were compared between the subgenogroup C2-like group and other subgenogroup groups by using Student's *t*-test with log_10_-transformed data. The p value < 0.05 is taken to indicate statistically significance.

### Nucleotide sequence accession numbers

The nucleotide sequences newly determined in this study have been submitted to the GenBank under the accession no. HM622381 to HM622392.

## Competing interests

The authors declare that they have no competing interests.

## Authors' contributions

YPH, TLL drafted the manuscript. YPH, WBF performed sequence and data analysis. TLL, YHT, CCH performed virus isolation, viral identification and neutralization test. LCH, YJC collected epidemiological information and edited the manuscript. JYY, FYC provided consultation and editing of the manuscript. HSW revised the manuscript. All authors read and approved the final manuscript.

## Supplementary Material

Additional file 1 **Phylogenetic analysis of enterovirus 71**. The phylogenetic tree was constructed by the neighbor-joining method with *MEGA *version 4 software, and the reliabilities indicated at the branch nodes were evaluated using 1,000 bootstrap replications. Only values of over 70% were shown. The prototype coxsackievirus A16 (CA16) G-10 strain was used as an out-group. The tree was drawn based on the 5'UTR (A), VP4 (B), VP2 (C), VP3 (D), VP1 (E), 2A (F), 2B (G), 2C (H), 3A (I), 3B (J), 3C (K), and 3D (L) region nucleotide sequences. Click here for file
