# Supplementary figures and images for: Genetic diversity and C2-like subgenogroup strains of enterovirus 71, Taiwan, 2008
Source: Virol J. 2010 Oct 20;7:277. doi: 10.1186/1743-422X-7-277 (PMC2975644; doi:10.1186/1743-422X-7-277)

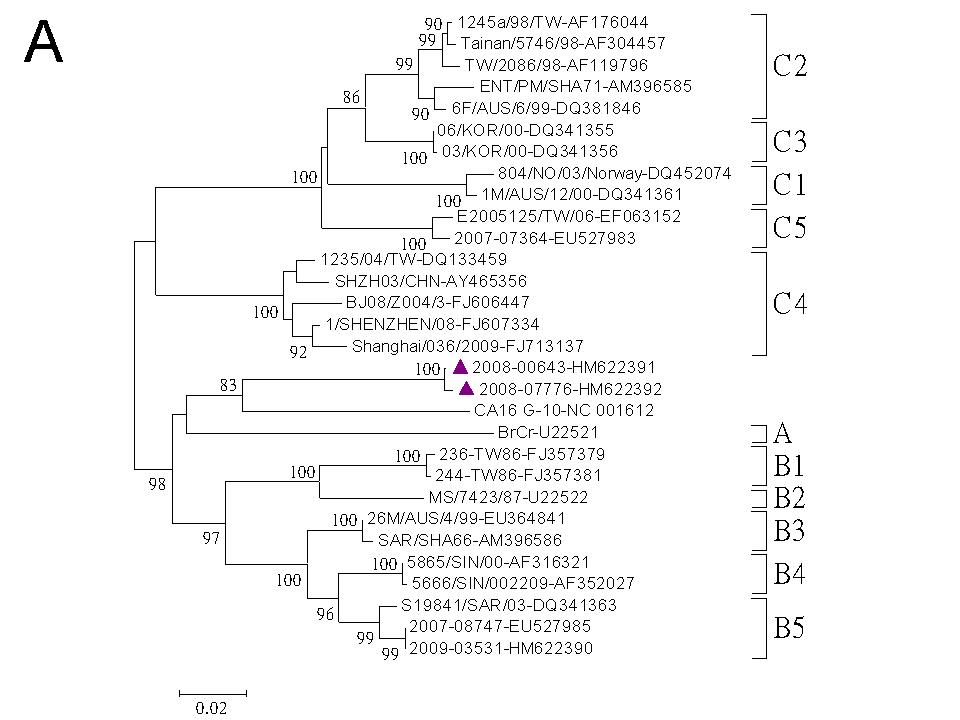


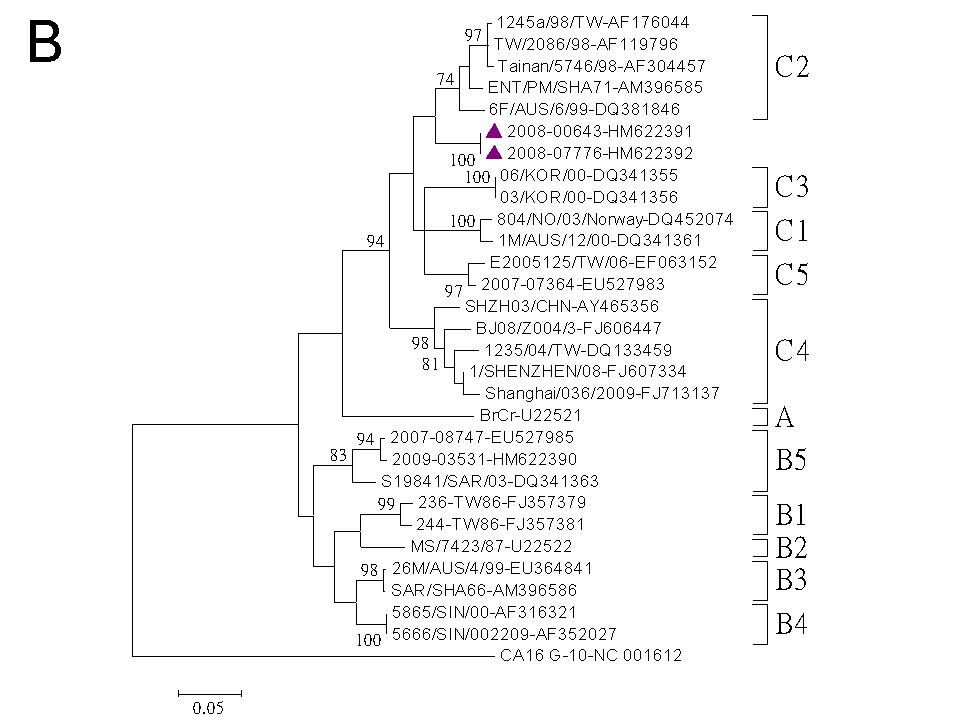


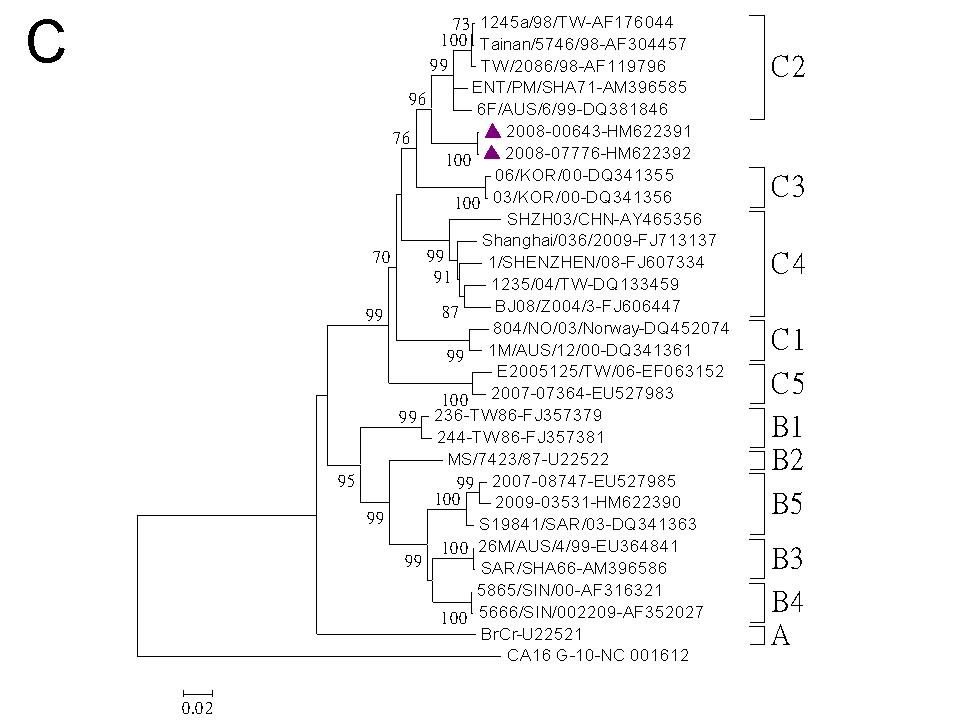


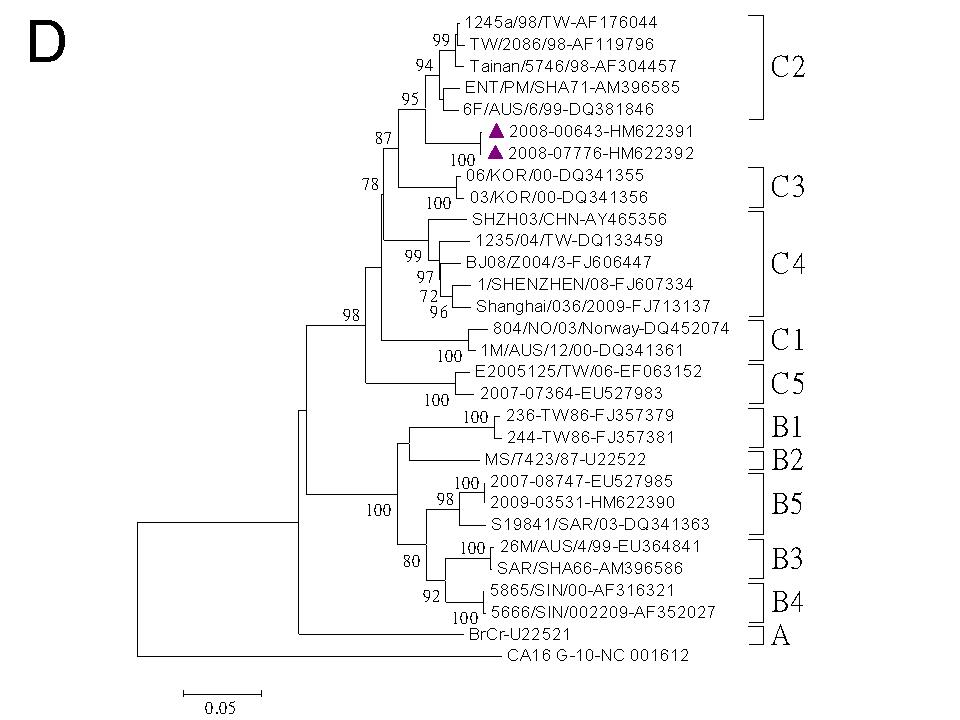


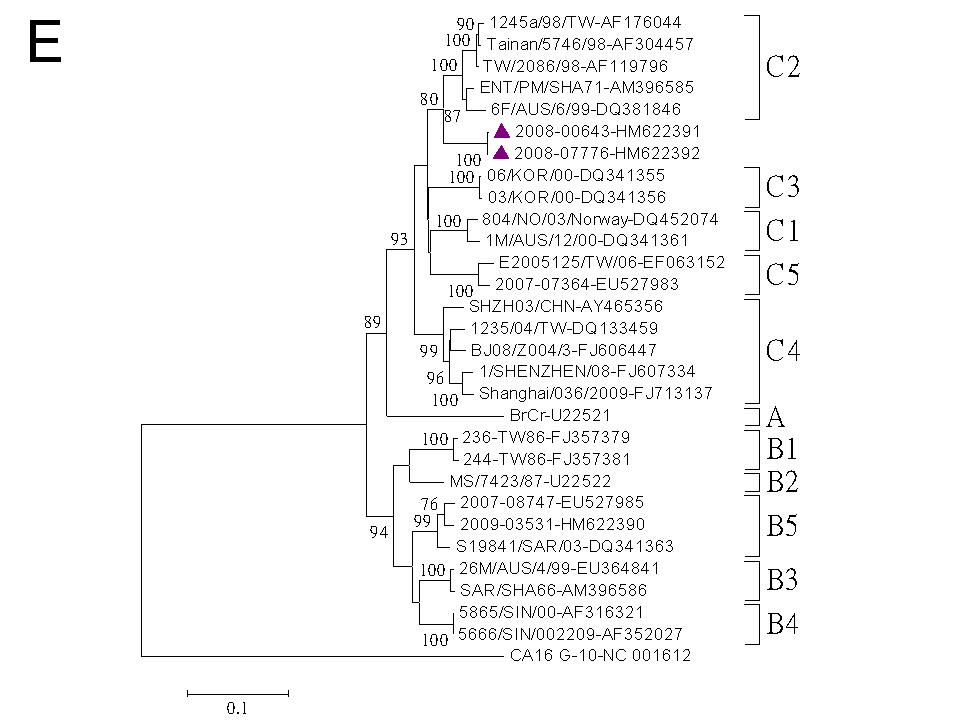


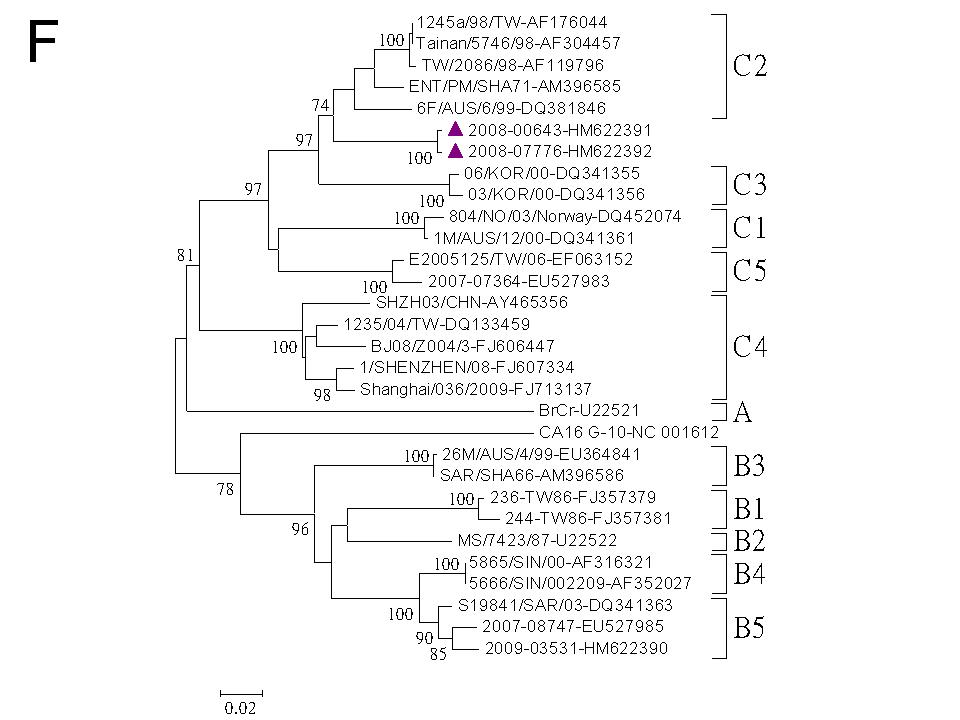


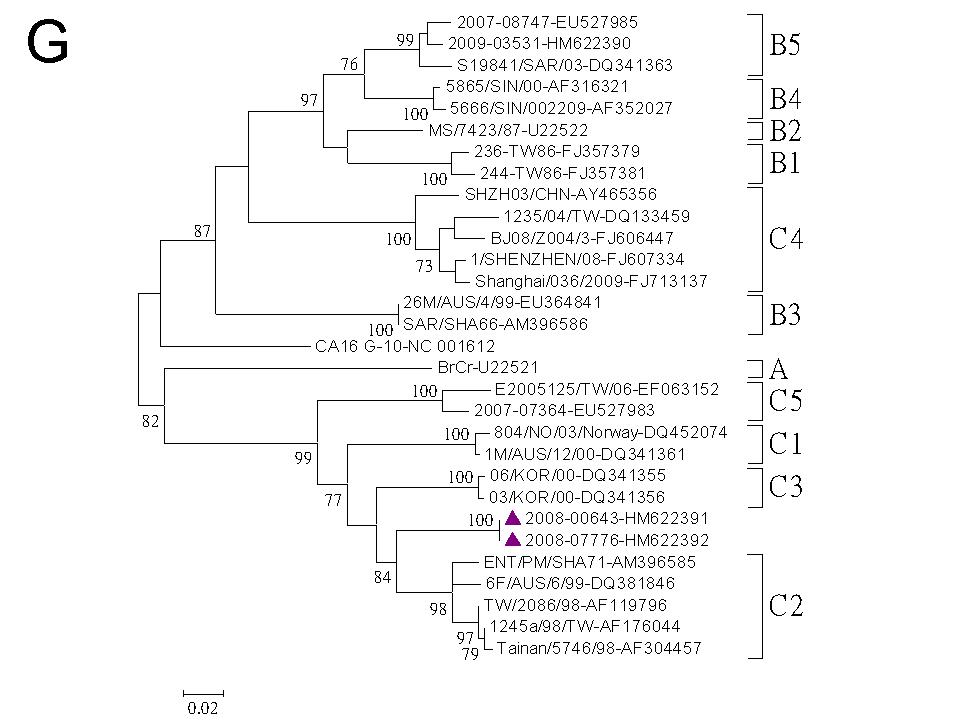


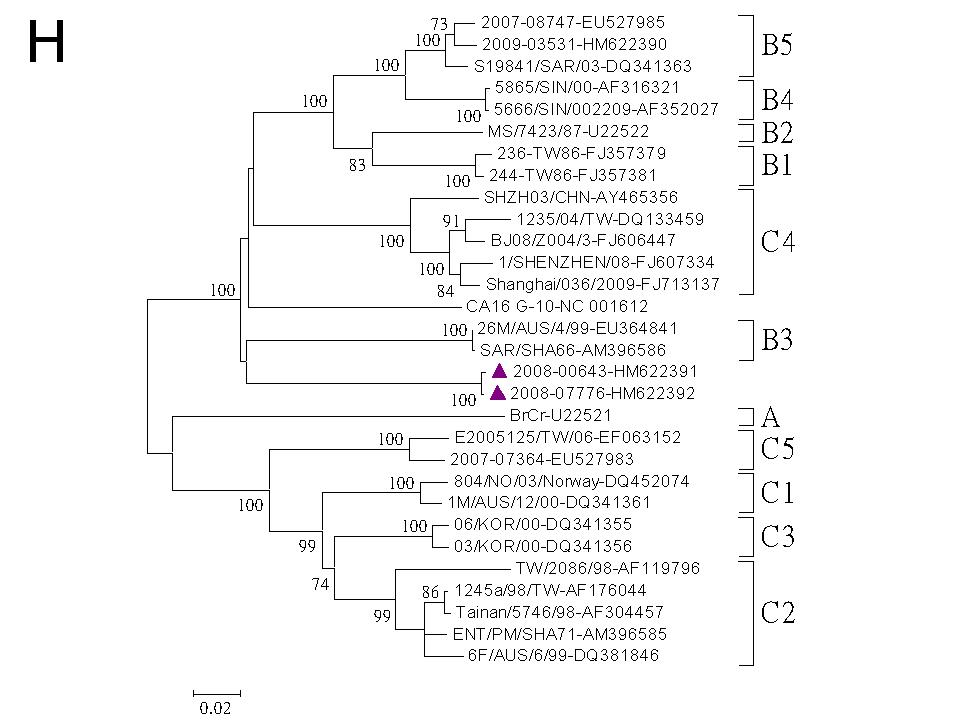


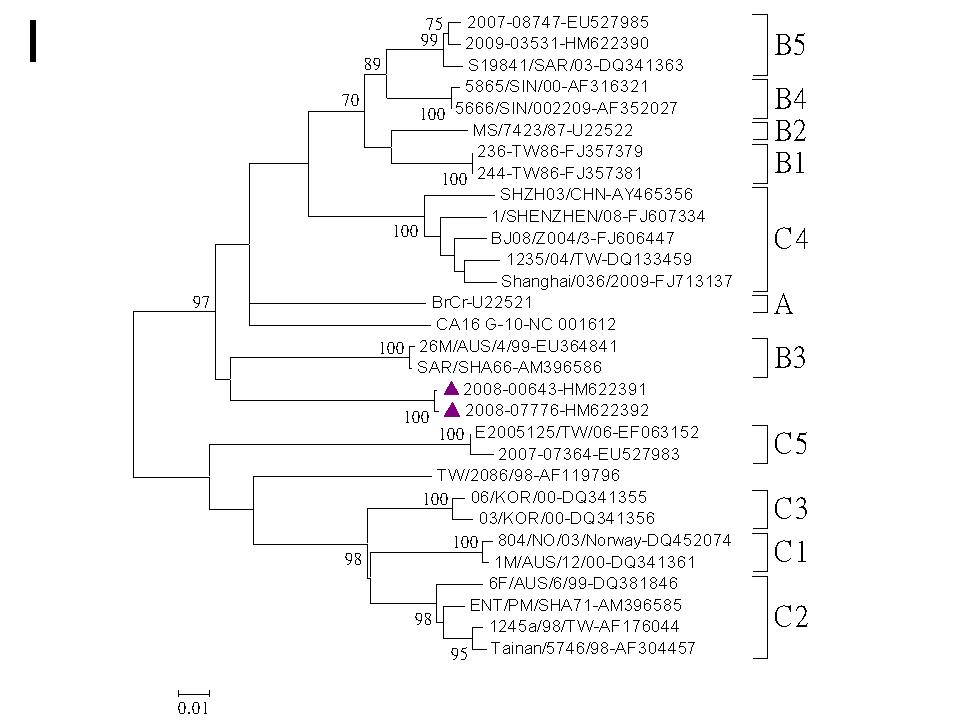


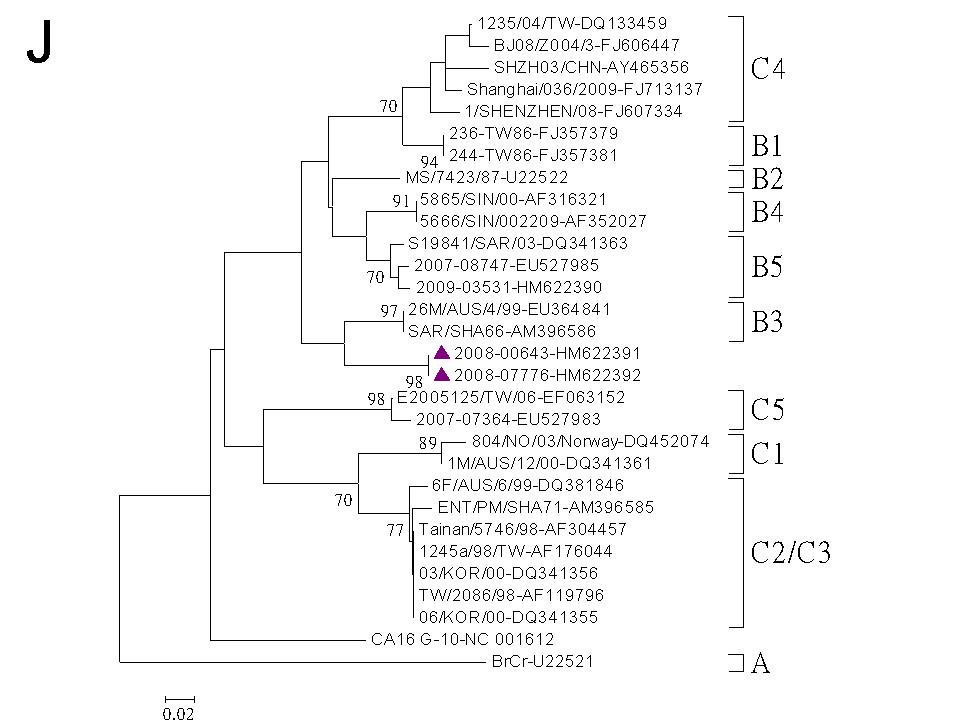


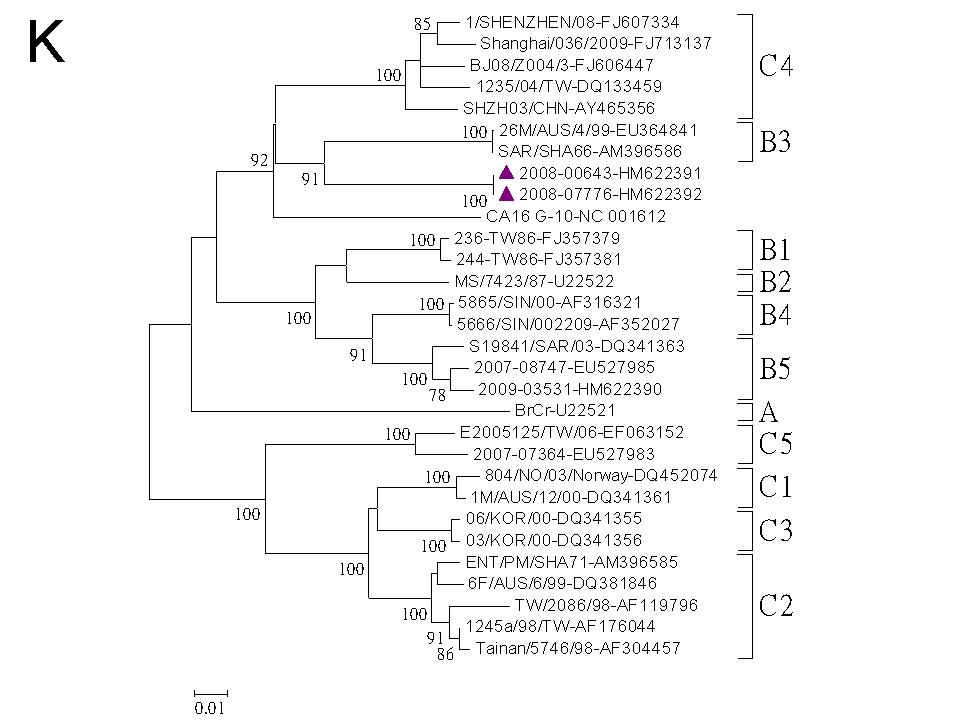


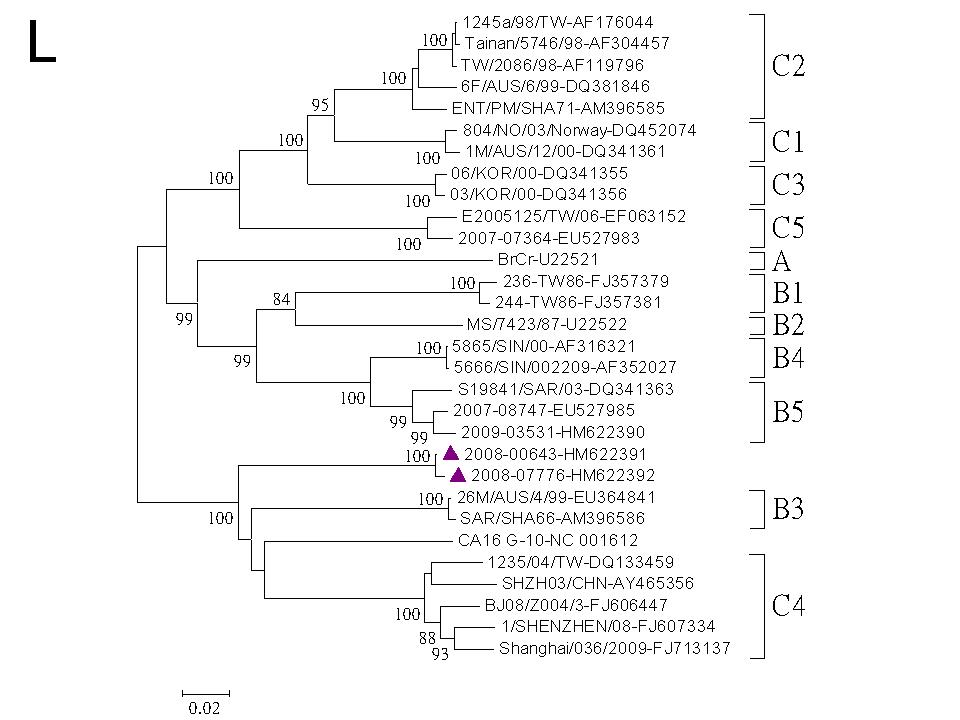

Supplement: Additional file 1 — Phylogenetic analysis of enterovirus 71. The phylogenetic tree was constructed by the neighbor-joining method with MEGA version 4 software, and the reliabilities indicated at the branch nodes were evaluated using 1,000 bootstrap replications. Only values of over 70% were shown. The prototype coxsackievirus A16 (CA16) G-10 strain was used as an out-group. The tree was drawn based on the 5'UTR (A), VP4 (B), VP2 (C), VP3 (D), VP1 (E), 2A (F), 2B (G), 2C (H), 3A (I), 3B (J), 3C (K), and 3D (L) region nucleotide sequences. [file 1743-422X-7-277-S1.DOC]
